# Splitting Teeth with the Mallet

**Published:** 1871-03

**Authors:** W. E. Driscoll


### SPLITTING TEETH WITH THE MALLET.

BY W. E. DRISCOLL.

At the late meeting of the Ohio Dental Society, Dr. Kelsey spoke of a case where two teeth were split by malleting in tin fillings, which was followed by a general expression of incredulity by the members of the society.

I had two cases about a year ago, that might be of interest in this connection.

The cases were quite similar, both in middle aged men; molars, with near half of crowns decayed away, with remaining portions quite strong; pulps devitalized, and in all respects, apparently, such cases as would be most proper for tin fillings. Used tin foil in cylinders, and condensed with a one ounce wood mallet, and with no more force than was agreeable to patient. Both were finished up nicely, and no indication of fracture or other undue violence could be detected.

Both returned within a few weeks after their respective operations, with crowns broken off very evenly all round near the gum. One broke off during mastication, the other "between meals."

Both parties disclaim any rough usage of the broken teeth. The roots not giving any trouble remain.

In consequence of these and other cases not quite so marked, I have since operated upon the principle that such bursting was to be carefully guarded against.

I am led to report these cases, by the surprise that was expressed at Dr. Kelsey's case; but not as an argument against the mallet, for I have been using for sometime, one of lead, weighing nine ounces, and my patients prefer it to the one of wood above mentioned, and that it condenses the gold better, a trial must convince any one.

